# Association of glycemic gap with stroke recurrence in patients with ischemic stroke

**DOI:** 10.1111/1753-0407.13432

**Published:** 2023-06-09

**Authors:** Kang Yuan, Mengdi Xie, Huajuan Hou, Jingjing Chen, Xinyi Zhu, Huaiming Wang, Xiaohao Zhang, Yi Xie, Min Wu, Rui Liu, Xinfeng Liu

**Affiliations:** ^1^ Department of Neurology, Nanjing Jinling Hospital Affiliated Hospital of Medical School, Nanjing University Nanjing China; ^2^ Department of Neurology, Jinling Hospital Nanjing Medical University Nanjing China; ^3^ Department of Neurology, Changhai Hospital Navy Medical University Shanghai China; ^4^ Department of Neurology The 80th Group Army Hospital of The People's Liberation Army Weifang China; ^5^ Department of Neurology, Nanjing First Hospital Nanjing Medical University Nanjing China

**Keywords:** A1C‐derived average glucose, glycemic gap, ischemic stroke, recurrence, A1C衍生的平均葡萄糖, 血糖间隙, 缺血性卒中, 复发

## Abstract

**Background:**

Glycemic gap, as a novel index of acute glycemic excursion, is associated with poor prognosis of different diseases. This study aimed to explore the association of the glycemic gap with long‐term stroke recurrence in patients with ischemic stroke.

**Methods:**

This study included patients with ischemic stroke from the Nanjing Stroke Registry Program. The glycemic gap was calculated by subtracting the estimated average blood glucose from the blood glucose at admission. Multivariable Cox proportional hazards regression analysis was performed to explore the association between the glycemic gap and the risk of stroke recurrence. The Bayesian hierarchical logistic regression model was used to estimate the effects of the glycemic gap on stroke recurrence stratified by diabetes mellitus and atrial fibrillation.

**Results:**

Among 2734 enrolled patients, 381 (13.9%) patients experienced stroke recurrence during a median follow‐up of 3.02 years. In multivariate analysis, glycemic gap (high group vs. median group) was associated with significantly increased risk for stroke recurrence (adjusted hazard ratio, 1.488; 95% confidence interval, 1.140–1.942; *p* = .003) and had varying effects on stroke recurrence depending on atrial fibrillation. The restricted cubic spline curve showed a U‐shaped relationship between the glycemic gap and stroke recurrence (*p* = .046 for nonlinearity).

**Conclusion:**

Our study found that the glycemic gap was significantly associated with stroke recurrence in patients with ischemic stroke. The glycemic gap was consistently associated with stroke recurrence across subgroups and had varying effects depending on atrial fibrillation.

## Graphical abstract

**Highlights**

- There was a U‐shaped relationship between glycemic gap and stroke recurrence during a long‐term follow‐up in a Chinese ischemic stroke registry.
- Clinical physicians should remain vigilant in the fluctuation of glucose level and use individualized glucose control strategies in patients with ischemic stroke.

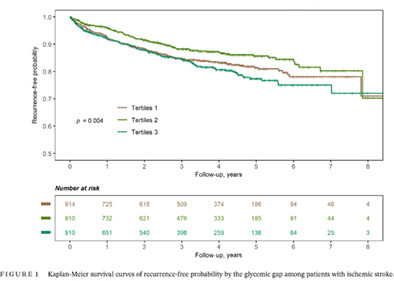

## 摘要

背景:血糖间隙作为一种新的急性血糖波动指标, 与不同疾病的不良预后有关。本研究旨在探讨缺血性脑卒中患者血糖间隙与长期卒中复发的关系。

方法:本研究纳入了来自南京卒中登记项目的缺血性卒中患者。血糖间隙通过从入院时的血糖中减去估计的平均血糖来计算。采用多变量Cox比例风险回归分析, 探讨血糖间隙与卒中复发风险之间的关系。采用贝叶斯分层logistic回归模型估计血糖间隙对糖尿病和房颤分层卒中复发的影响。

结果:在2734例入组患者中, 381例(13.9%)患者在3.02年的中位随访期间出现卒中复发。在多变量分析中, 血糖间隙(高组vs中值组)与卒中复发风险显著增加相关(校正危险比, 1.488;95%置信区间为1.140 ~ 1.942;P = 0.003), 心房颤动对卒中复发有不同的影响。限制三次样条曲线显示血糖间隙与卒中复发呈U型关系(p =0.0046表示非线性)。

结论:我们的研究发现, 血糖间隙与缺血性卒中患者的卒中复发有显著关系。在各个亚组中, 血糖间隙始终与卒中复发相关, 并且根据房颤的不同有不同的影响。

## 1 INTRODUCTION

Stroke is a leading cause of death and disability worldwide. Despite advances in secondary prevention strategies,^1^ 7.9% of stroke survivors will suffer stroke recurrence within 2 years.^2^ Diabetes mellitus (DM) and atrial fibrillation (AF) are major risk factors for ischemic stroke.^3^, ^4^ Previous studies suggested that the prevalence of both DM and AF had been appreciably elevated in recent years.^5^, ^6^ Epidemiological data revealed that DM was associated with vascular complications, and the coexistence of AF would increase the risk of ischemic stroke.^7^ Hence, a comprehensive understanding is needed to elucidate how the risk of stroke recurrence may interact with DM and AF in patients with ischemic stroke.

The association between glycemic control and stroke risk has been recently investigated in patients with DM and AF, with poor glycemic control associated with an increased risk of stroke.^8^, ^9^ However, inconsistent results have been found between blood glucose and clinical outcomes of ischemic stroke.^10^, ^11^ Blood glucose could fluctuate with physical conditions at admission, while glycated hemoglobin (HbA1c) is relatively stable.^12^ The glycemic gap is a surrogate marker calculated by subtracting the HbA1c‐derived estimated average glucose from blood glucose and may reflect glycemic excursion in the acute phase.^13^ Prior reports revealed that the glycemic gap was associated with left ventricular systolic dysfunction in patients with myocardial infarction^14^ and contributed to poor outcomes in patients with spontaneous intracerebral hemorrhage.^15^ Nevertheless, studies evaluating the relationship between the glycemic gap and stroke recurrence regarding DM and AF remain limited at present. Hence, this study aimed to explore the correlation between glycemic gap and long‐term stroke recurrence in patients with ischemic stroke.

## 2 METHODS

The data that support the findings of this study are available from the corresponding author on reasonable request.

### 2.1 Study populations

This study comprised consecutive participants from the Nanjing Stroke Registry Program between January 2013 and December 2019. The Nanjing Stroke Registry Program was an ongoing, prospective observational project and a hospital‐based stroke registry conducted in China. Details of the Nanjing Stroke Registry Program had been published previously.^16^ The Ethics Review Board of Jinling Hospital approved this study (approval number 2010NLY‐018). Written informed consents were obtained from all registered patients.

Patients were included if they (a) had ischemic stroke diagnosed within 7 days after stroke onset, (b) were aged ≥18 years, and (c) had brain computed tomography or magnetic resonance imaging examinations right before or during hospitalization. Patients were excluded if they (a) had no measurement of glucose concentration or HbA1c concentration, and (b) were diagnosed with pre‐existing hypoglycemia (<3.9 mmol/L) and hyperosmolar hyperglycemic states (>33.3 mmol/L).

### 2.2 Covariates

Demographics, vascular risk factors, radiological images, laboratory findings, stroke characteristics, prior antidiabetic agents, and medications at discharge were all collected during hospitalization. DM was defined according to the American Diabetes Association criteria for the diagnosis of diabetes.^17^ AF was defined as previously or newly diagnosed AF with the 12‐lead electrocardiogram or Holter monitoring after admission.^18^ Stroke characteristics included stroke subtype classified according to the trial of ORG 10172 in Acute Stroke Treatment classification^19^ and stroke severity assessed by the National Institutes of Health Stroke Scale (NIHSS).^20^ Education years, smoking status, and alcohol consumption were investigated with face‐to‐face questionnaires. Smoking status was classified as never, former, and current smokers according to the consumption of cigarettes.

### 2.3 Glycemic gap calculation

Blood samples were routinely obtained within 24 h after admission. The estimated average blood glucose level (eAG) was derived from the following equation: eAG (mg/dL) = 28.7 × HbA1c (%) − 46.7. The glycemic gap was calculated by subtracting eAG levels from the blood glucose concentrations at admission.

### 2.4 Follow‐up and outcomes

Patients were followed in person or by structured telephone interviews by well‐trained stroke investigators at 3, 6, and 12 months and annually after the index stroke. Detailed information about mortality, recurrences, clinical scores, and revisions of the initial diagnosis regarding the index stroke were recorded at each follow‐up.

The primary end point was the long‐term fatal or nonfatal recurrent stroke, which was defined as a new neurological deficit or the deterioration of previous deficits that met the diagnostic criterion of ischemic or hemorrhagic stroke.^21^ The length of hospitalizations and other endpoints were also recorded: (a) all‐cause mortality confirmed by medical records, death certificates, or other available data at each follow‐up; and (b) favorable outcome defined as modified Rankin Scale (mRS) scores of 0–2 at 90 days.

### 2.5 Statistical analysis

Baseline characteristics were presented as mean (SD) or median (interquartile range) for continuous variables and numbers (percentage) for categorical variables. We divided patients into different groups according to glycemic gap tertiles. We used the *χ* ^2^ test or Fisher exact test to compare categorical variables and Student *t* test or Mann‐Whitney *U*‐test to compare continuous variables as appropriate. We used the multiple imputation method with chain equations to deal with missing values.

We used the Cox proportional hazards regression analysis to calculate the hazard ratio (HR) and the 95% confidence interval (CI) for the association of the glycemic gap with stroke recurrence and all‐cause mortality. We also fitted multivariable logistic regression models to analyze the relationship between the glycemic gap and favorable outcome. We plotted the probabilities of favorable outcome according to the glucose and glycemic gap in the three‐dimensional distribution surface diagram. We selected covariates based on comparative effectiveness researches and clinical experience. Model 1 was an unadjusted model. Model 2 was adjusted for age, sex, hypertension, DM, dyslipidemia, coronary heart disease, smoking status, alcohol consumption, stroke etiology, and education years. Model 3 was additionally adjusted for body mass index, NIHSS, hemoglobin, total cholesterol, triglyceride, high‐density lipoprotein, low‐density lipoprotein, prior antidiabetic agents, and the usage of antiplatelet drugs, anticoagulants, antihypertensive drugs and hypoglycemic agents at discharge.

We presented the risk of stroke recurrence stratified to glycemic gap tertiles in Kaplan–Meier curves and tested the proportional‐hazards assumption with the Schoenfeld residuals. We used the restricted cubic splines with four knots (at the 5th, 35th, 65th, and 95th percentiles) to evaluate the nonlinearity of the association between the glycemic gap and stroke recurrence.^22^ We used the fractional polynomial model to calculate the optimal glycemic gap level, which was the smallest HR for stroke recurrence. On a more granular level, we performed subgroup analyzes to investigate the association of the glycemic gap with stroke recurrence stratified by DM and AF. We also employed the Bayesian hierarchical logistic regression model to estimate the effects of the glycemic gap on stroke recurrence separately for AF and calculated the substance level via the posterior predictive distributions. We also performed the competing risk analysis by treating mortality as the competing risk using the Fine and Gray method. Further details were provided in the Supplementary Methods.

Statistical analyses were implemented in R (version 4.2.1, R Core Team, Vienna, Austria) and Python (version 3.7.10, Python Software Foundation). A two‐sided *p* value <.05 was considered to be statistically significant.

## 3 RESULTS

### 3.1 Patient characteristics

A total of 2734 patients (median age, 62 [54, 70] years; male sex, 70.4%) with ischemic stroke were included in this study after excluding 1092 patients with missing glucose or HbA1c values, 92 patients with missing follow‐up information and 57 patients with preexisting hypoglycemia or hyperosmolar hyperglycemic states (Figure S1). The baseline characteristics according to tertiles of glycemic gaps were presented in Table 1. Patients with higher glycemic gaps had higher levels of glucose and lower levels of HbA1c (*p* < .001). There were significant differences in age, body mass index, stroke severity, the level of white blood count, triglyceride, high density lipoprotein, alanine aminotransferase, and the proportion of DM, AF, stroke subtypes, prior antidiabetic agents, and the usage of antiplatelet drugs, statins, and hypoglycemic agents at discharge among these groups (*p* < .05). The proportions of missing values were presented in Table S1. The baseline characteristics between patients excluded due to missing HbA1c levels and those included in the study were comparable except for age, the proportion of DM and white blood cell counts (Table S2).

**TABLE 1:** Baseline characteristics of patients with ischemic stroke according to tertiles of glycemic gaps.

	Total	Tertiles 1 (≤−34.48 mg/dL)	Tertiles 2 (−34.48 to −17.60 mg/dL)	Tertiles 3 (>−17.60 mg/dL)	
Characteristics	*N* = 2734	*N* = 914	*N* = 910	*N* = 910	*p* value
Age, years	61.7 (11.1)	62.0 (10.8)	60.9 (11.1)	62.3 (11.5)	.018
Male, *n* (%)	1924 (70.4)	670 (73.3)	637 (70.0)	617 (67.8)	.305
BMI, kg/m^2^	24.7 (3.3)	24.9 (3.4)	24.7 (3.3)	24.5 (3.2)	.018
Baseline NIHSS, score	4 [2, 9]	3 [1, 6]	4 [1, 7]	6 [2, 13]	<.001
Hypertension, *n* (%)	2007 (73.4)	656 (71.8)	664 (73.0)	687 (75.5)	.185
Diabetes mellitus, *n* (%)	1062 (38.8)	571 (62.5)	188 (20.7)	303 (33.3)	<.001
Dyslipidemia, *n* (%)	862 (31.5)	294 (32.2)	279 (30.7)	289 (31.8)	.614
Atrial fibrillation, *n* (%)	296 (10.8)	72 (7.9)	80 (8.8)	144 (15.8)	<.001
Coronary heart disease, *n* (%)	261 (9.5)	96 (10.5)	72 (7.9)	93 (10.2)	.094
Smoking status, *n* (%)					.206
Never	1288 (47.1)	403 (44.1)	424 (46.6)	461 (50.7)	
Former	254 (9.3)	81 (8.9)	92 (10.1)	81 (8.9)	
Current	1192 (43.6)	430 (47.0)	394 (43.3)	368 (40.4)	
Alcohol consumption, *n* (%)	585 (21.4)	171 (18.7)	204 (22.4)	210 (23.1)	.732
Education, years					.658
0–6	816 (29.8)	267 (29.2)	281 (30.9)	268 (29.5)	
6–9	1304 (47.7)	439 (48.0)	420 (46.2)	445 (48.9)	
9–12	319 (11.7)	110 (12.0)	110 (12.1)	99 (10.9)	
>12	295 (10.8)	98 (10.7)	99 (10.9)	98 (10.8)	
Stroke subtypes, *n* (%)					<.001
LAA	991 (36.2)	374 (40.9)	317 (34.8)	300 (33.0)	
CE	354 (12.9)	74 (8.1)	102 (11.2)	178 (19.6)	
SAA	530 (19.4)	203 (22.2)	182 (20.0)	145 (15.9)	
SOE	298 (10.9)	89 (9.7)	113 (12.4)	96 (10.5)	
SUE	561 (20.5)	174 (19.0)	196 (21.5)	191 (21.0)	
Laboratory data					
White blood cell count, 10^9^/L	7.0 [5.8, 8.6]	7.0 [5.8, 8.3]	6.8 [5.7, 8.2]	7.4 [5.9, 9.4]	<.001
Hemoglobin, g/L	140 [129, 151]	139 [129, 150]	141 [130, 151]	140 [127, 151]	.101
TC, mmol/L	4.3 (1.2)	4.4 (1.2)	4.3 (1.2)	4.3 (1.1)	.637
TG, mmol/L	1.4 [1.0, 1.9]	1.4 [1.1, 1.9]	1.4 [1.0, 1.9]	1.3 [1.0, 1.8]	<.001
HDL, mmol/L	1.1 (0.4)	1.0 (0.3)	1.1 (0.3)	1.1 (0.5)	<.001
LDL, mmol/L	2.7 (1.0)	2.7 (1.0)	2.7 (1.0)	2.6 (0.9)	.284
Glucose, mmol/L	6.4 (2.5)	6.2 (2.3)	5.5 (1.6)	7.4 (3.0)	<.001
Creatinine, μmmol/L	68.7 (29.3)	70.6 (35.9)	66.9 (19.1)	68.5 (30.1)	.025
HbA1c, %	6.6 (1.7)	7.6 (1.9)	6.0 (1.0)	6.1 (1.4)	<.001
ALT, U/L	17 [13, 26]	17 [12, 26]	17 [13, 25]	19 [13, 28]	<.001
Prior antidiabetic agents, *n* (%)					<.001
None	2175 (79.6)	612 (67.0)	822 (90.3)	741 (81.4)	
OHA	184 (6.7)	88 (9.6)	39 (4.3)	57 (6.3)	
Insulin	142 (5.2)	87 (9.5)	11 (1.2)	44 (4.8)	
Both	233 (8.5)	127 (13.9)	38 (4.2)	68 (7.5)	
Medications at discharge, *n* (%)					
Antiplatelet drugs	2481 (90.7)	857 (93.8)	843 (92.6)	781 (85.8)	<.001
Anticoagulants	183 (6.7)	48 (5.3)	62 (6.8)	73 (8.0)	.302
Statins	2610 (95.5)	894 (97.8)	883 (97.0)	833 (91.5)	<.001
Antihypertensive drugs	1186 (43.4)	382 (41.8)	395 (43.4)	409 (44.9)	.508
Hypoglycemic agents	877 (32.1)	499 (54.6)	139 (15.3)	239 (26.3)	<.001
Length of hospitalization, days	11 [8, 14]	10 [8, 14]	10 [8, 14]	12 [9, 16]	<.001

Abbreviations: ALT, alanine aminotransferase; BMI, body mass index; CE, cardioembolism; HbA1c, glycated hemoglobin; HDL, high‐density lipoprotein; LAA, large‐artery atherosclerosis; LDL, low‐density lipoprotein; NIHSS, National Institute of Health Stroke Scale; OHA, oral hypoglycemic agents; SAA, small‐vessel occlusion; SOE, stroke of other determined etiology; SUE, stroke of undetermined etiology; TC, total cholesterol; TG, triglyceride.

### 3.2 Glycemic gaps and stroke recurrence

**FIGURE 1:**
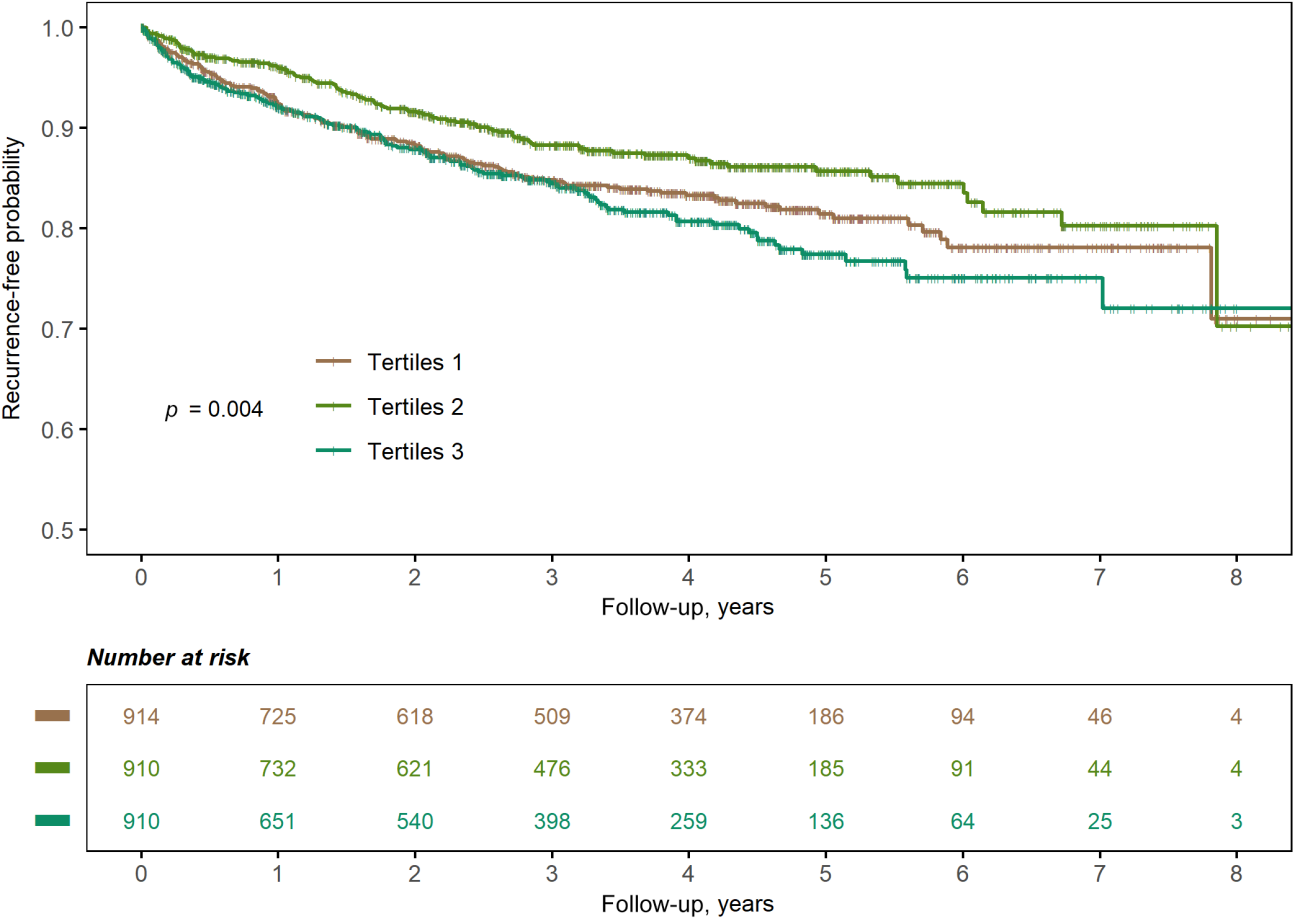
Kaplan‐Meier survival curves of recurrence‐free probability by the glycemic gap among patients with ischemic stroke.

**FIGURE 2:**
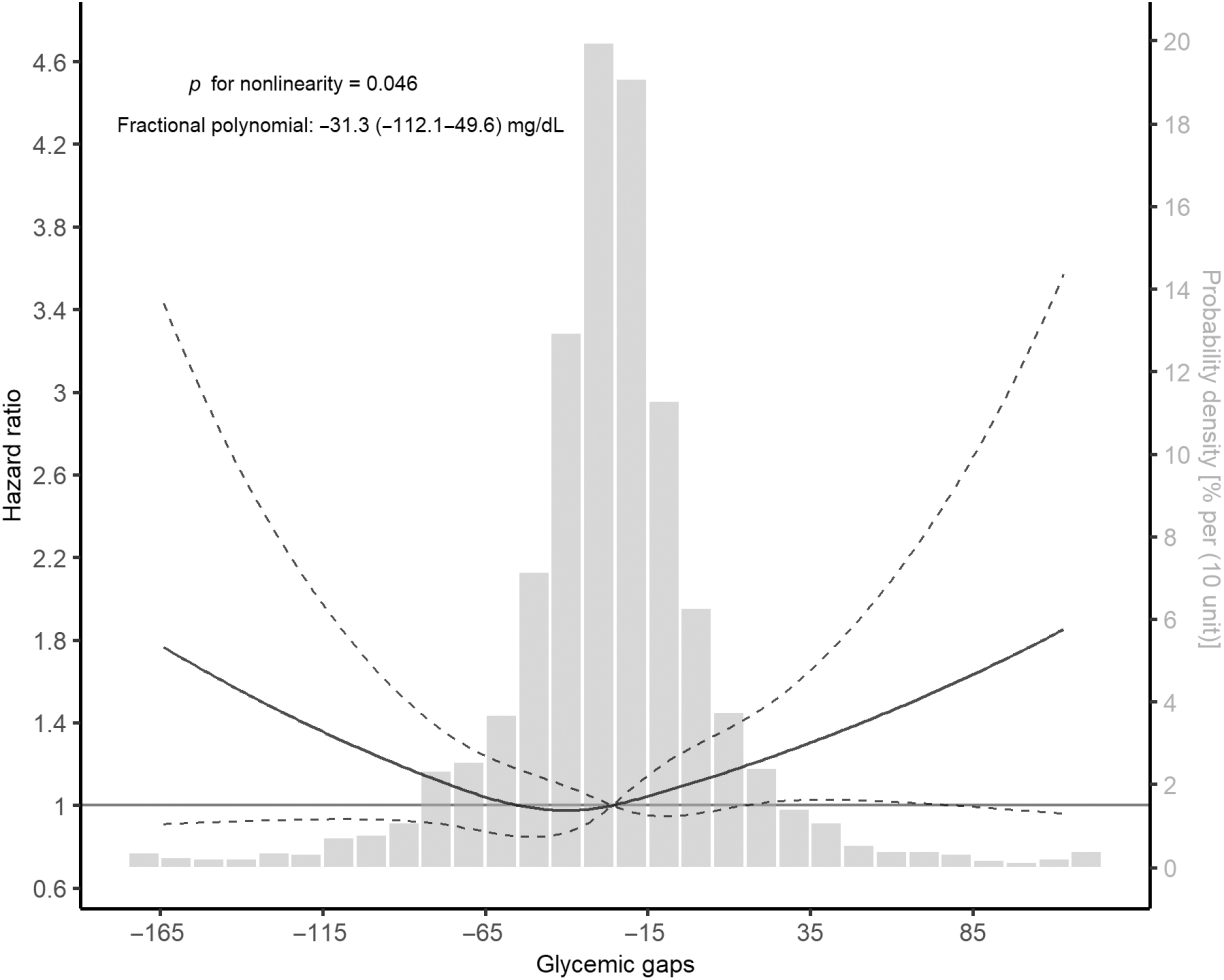
Optimal ranges of the glycemic gap for the risk of stroke recurrence. The optimal range of the glycemic gap was calculated from the linear‐quadratic and fractional polynomial models and nonlinearity was evaluated with the restricted cubic spline curve. The solid line presented the hazard ratio and the dashed lines presented the 95% confidence interval.

During a median follow‐up of 3.02 (1.11–4.58) years, 381 (13.9%) patients experienced stroke recurrence. Univariable predictors of stroke recurrence were age (HR, 1.014; 95% CI, 1.005–1.024, *p* = .010), hypertension (HR, 1.351; 95% CI, 1.058–1.725, *p* = .016), AF (HR, 1.509; 95% CI, 1.124–2.027, *p* = .006), smoking status (current vs. never: HR, 1.239; 95% CI, 1.003–1.531, *p* = .047), alcohol consumption (HR, 0.626; 95% CI, 0.453–0.865, *p* = .004), stroke subtype (small‐vessel occlusion vs. large‐artery atherosclerosis: HR, 0.635; 95% CI, 0.464–0.867, *p* = .004), DM (HR, 1.225; 95% CI, 1.001–1.501; *p* = .049), and the usage of hypoglycemic agents at discharge (HR, 1.249; 95% CI, 1.014–1.5308 *p* = .036; Table S3). The Kaplan–Meier curve showed that the medium tertile of the glycemic gap had the lowest risk of stroke recurrence (log‐rank *p* = .004, Figure 1). The restricted cubic spline curve showed a nonlinear relationship between the glycemic gap and stroke recurrence (*p* = .046 for nonlinearity, Figure 2). The best fitting fractional polynomial model included the scaled terms of glycemic gap^2^ and glycemic gap^2^ * ln(glycemic gap), and the optimal range of glycemic gap was −31.3 (−112.1 to 49.6) mg/dL in the fractional polynomial model.

### 3.3 Multivariable analysis for clinical outcomes

In multivariable analyses, glycemic gaps (high group vs. median group) were associated with a significantly increased risk for stroke recurrence in model 1 (HR, 1.527; 95% CI, 1.183–1.970; *p* = .001), model 2 (HR, 1.488; 95% CI, 1.140–1.942; *p* = .003), and model 3 (HR,1.488; 95% CI, 1.140–1.942; *p* = .003; Table 2). Glycemic gaps were also associated with an increased risk for all‐cause mortality in model 1 (HR, 2.304; 95% CI, 1.739–3.052; *p* <0.001), model 2 (HR, 1.936; 95% CI, 1.454–2.578; *p* < .001), but not model 3 (HR, 1.327; 95% CI, 0.976–1.806; *p* = .071; Table S4). The distribution of mRS scores at 90 days according to glycemic gap tertiles showed that patients with higher glycemic gaps had lower proportions of favorable outcome (Figure S2). The association remained significant in model 1 (odds ratio [OR], 0.445; 95% CI, 0.351–0.562; *p* < .001), model 2 (OR, 0.491; 95% CI, 0.383–.628; *p* < .001), but not in model 3 (OR, 0.881; 95% CI, 0.659–1.177; *p* = .391; Table S5). The three‐dimensional distribution surface diagram showed that favorable outcome probability was reduced both with the decreases of glycemic gaps and glucose concentrations (Figure S3). Furthermore, the association between glycemic gap and stroke recurrence remained significant in competing risk analyzes (HR, 1.471; 95% CI, 1.128–1.919; *p* = .004; Table S6).

**TABLE 2:** Hazard ratios for stroke recurrence according to the glycemic gap.

		Model 1		Model 2		Model 3	
Variables	No. of events (%)	HR (95% CI)	*p* value	HR (95% CI)	*p* value	HR (95% CI)	*p* value
Tertiles 1	139/914 (15.2)	1.329 (1.03–1.715)	.029	1.240 (0.945–1.625)	.120	1.198 (0.911–1.576)	.197
Tertiles 2	103/910 (11.3)	Reference		Reference		Reference	
Tertiles 3	139/910 (15.3)	1.527 (1.183–1.970)	.001	1.488 (1.148–1.928)	.003	1.488 (1.140–1.942)	.003

Abbreviations: CI, confidence interval; HR, hazard ratio; NIHSS, National Institute of Health Stroke Scale.

*Note*: Model 1: unadjusted model. Model 2: adjusted by age, sex, hypertension, diabetes mellitus, atrial fibrillation, dyslipidemia, coronary heart disease, smoking status, alcohol consumption, stroke etiology, education years. Model 3: adjusted for covariates in model 2 and body mass index, NIHSS score, hemoglobin, total cholesterol, triglyceride, high‐density lipoprotein, low‐density lipoprotein, prior antidiabetic agents, and the usage of antiplatelet drugs, anticoagulants, antihypertensive drugs, and hypoglycemic agents at discharge.

### 3.4 Subgroup analysis

**FIGURE 3:**
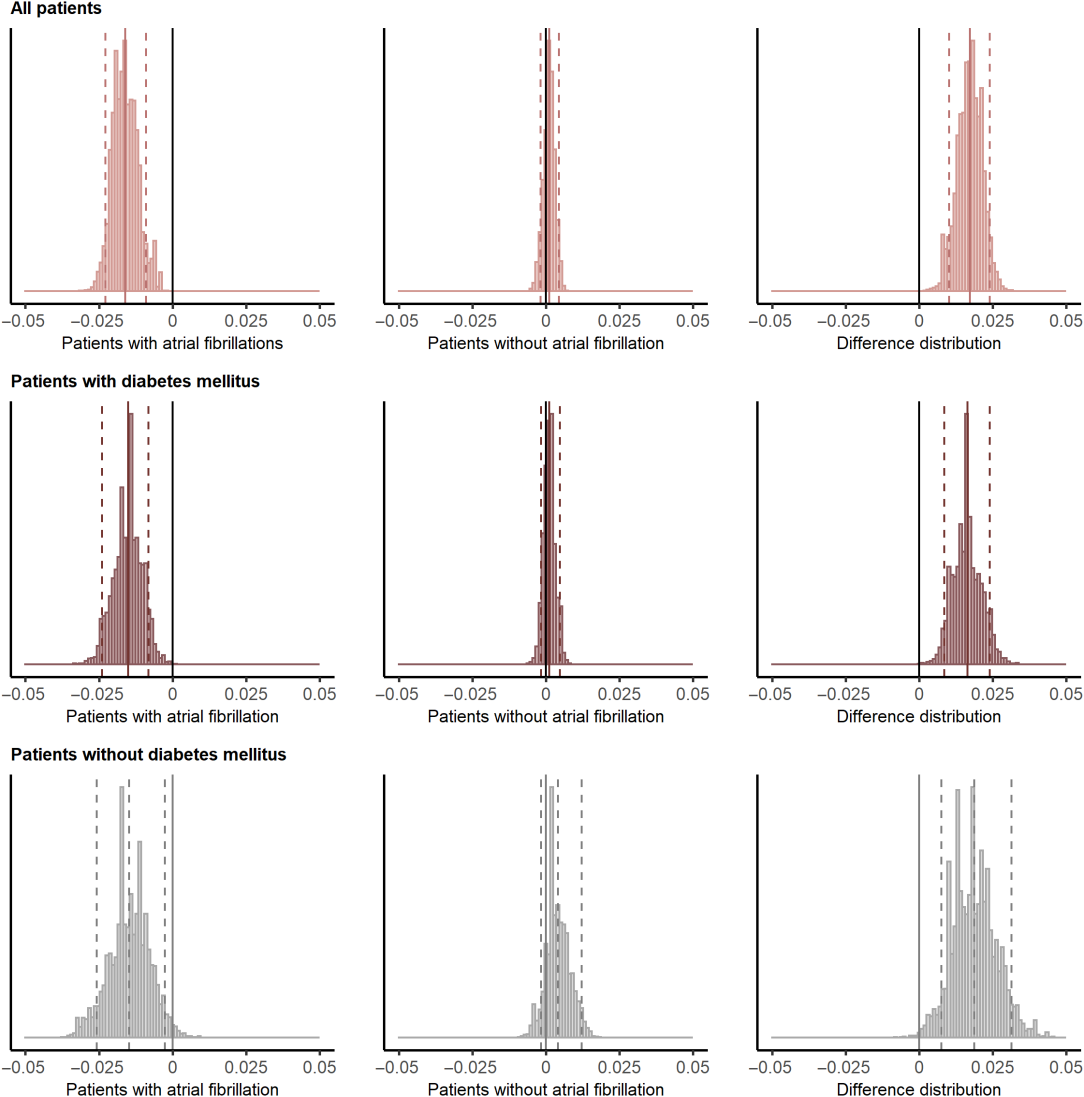
Bayesian hierarchical logistic regression modeling: glycemic gap effects on stroke recurrence depending on atrial fibrillation. The left column presented the effects of the glycemic gap on stroke recurrence in patients with atrial fibrillation, and the middle column presented the effects in patients without atrial fibrillation. The dashed lines in the right column presented the differences of the posteriors and the corresponding 90% highest probability density interval. The differences could be substantial when the solid line (the reference level of 0) were not included in the range of dashed lines.

Subgroup analysis for stroke recurrence according to DM and AF was shown in Figure S4 and Table S7. The association between the glycemic gap and stroke recurrence was significant in the patients without DM (tertile 3 vs. tertile 2: HR, 1.490; 95% CI, 1.080–2.056; *p* = .015) but not in patients with DM (tertile 3 vs. tertile 2: HR, 1.493; 95% CI, 0.904–2.465; *p* = .117). Higher glycemic gaps were associated with a higher risk of stroke recurrence in patients with non‐AF (tertile 3 vs. tertile 2: HR, 1.512; 95% CI, 1.143–2.001; *p* = .004), whereas lower glycemic gaps were associated with a higher risk in patients with AF (tertile 1 vs. tertile 2: HR, 4.403; 95% CI, 1.742–11.127; *p* = .002; Table S7). Moreover, glycemic gap had varying effects depending on AF in all patients (difference of posterior distributions: mean, 0.017; 90% highest probability density interval [HPDI], 0.010–0.024; not intersecting zero; upper row in Figure 3), patients with DM (difference of posterior distributions: mean, 0.016; 90% HPDI, 0.008–0.024; not intersecting zero; middle row), and patients without DM (difference of posterior distributions: mean, 0.019; 90% HPDI, 0.008–0.031; not intersecting zero; bottom row).

## 4 DISCUSSION

In the present study, we revealed a significant association between the glycemic gap and stroke recurrence in patients with ischemic stroke over a long‐term follow‐up period. The association between the glycemic gap and stroke recurrence remained significant after adjusting for covariates suggested to be associated with stroke recurrence. Furthermore, we found that the association was U shaped, and the optimal range of the glycemic gap related to the minimum recurrence risk was around −31.3 mg/dL. Finally, the glycemic gap was consistently associated with stroke recurrence across different subgroups and had varying effects depending on AF.

Previous studies regarding blood glucose and stroke recurrence in patients with ischemic stroke remain controversial. In the post hoc analysis of the CHANCE (Clopidogrel in High‐Risk Patients with Acute Nondisabling Cerebrovascular Events) trial, patients with impaired fasting glucose and DM were at higher risk of stroke after a minor stroke or transient ischemic attack, and there was a weak J‐shaped relationship between blood glucose and the risk of stroke.^23^ However, the SHINE (Stroke Hyperglycemia Insulin Network Effort) trial indicated that the influence of background glucose levels, such as preexisting abnormalities of glucose metabolism and stress‐induced hyperglycemia were important factors.^24^ Previous studies suggested that stress‐induced hyperglycemia or undiagnosed DM had a higher risk of vascular events than those with normal glucose levels.^25^ The glycemic gap could better reflect the effects of acute glycemic changes on the prognosis of ischemic stroke and may be more convenient than stress hyperglycemia ratio for a wider range of values.^26^ To the best of our knowledge, this was the first study to explore the association between the glycemic gap and the long‐term risk of stroke recurrence in patients stratified by DM and AF.

In line with previous studies, 13.9% of patients in our study experienced stroke recurrence after a median follow‐up of 3 years.^27^ Our study revealed a U‐shaped relationship between the glycemic gap and stroke recurrence. In the ACROSS‐China registry, Zhu et al found a J‐shaped relationship between glucose‐to‐HbA1c ratio and stroke recurrence in nondiabetic patients with ischemic stroke.^28^ Guo et al conducted a retrospective analysis in intensive care units and found a U‐shaped association between the glycemic gap and mortality in patients with critical illness. Subgroup analysis revealed that the association between glycemic gap and stroke recurrence was significant in DM and marginally significant in patients without DM, which might indicate that both uncontrolled DM and stress‐induced hyperglycemia in patients without DM could induce the development of stroke recurrence. Besides, the rates of mortality (11.0%)^2^ and favorable functional outcome (81.6%)^29^ were similar to previous reports, and a higher glycemic gap was related to an increased risk of mortality and a reduced likelihood of favorable functional outcome. Roberts et al suggested that stress hyperglycemia was associated with poor functional outcome in patients with ischemic stroke.^30^ Another prospective study indicated that a higher quartile of stress hyperglycemia was related to a higher risk of all‐cause death after stroke.^31^

The potential mechanisms of the association between the glycemic gap and stroke recurrence might be explained as follows. First, high glycemic gaps, which reflected relative hyperglycemia, may promote the release of inflammatory and vasoconstrictive factors and trigger the inflammatory and neurohormonal response to acute ischemic stroke.^32^ Second, glucose fluctuations may induce a prothrombotic state via vascular inflammation, vascular permeability, nitric oxide inactivation, and the production of reactive oxygen species.^33^ Third, hyperglycemia can enhance endothelial dysfunction and oxidative stress, which are important factors in cerebrovascular diseases.^34^, ^35^

Previous studies indicated that AF accounted for 10.8% of ischemic stroke and was the main source of cardioembolic stroke.^27^ The Bayesian hierarchical logistic regression model showed that glycemic gap had varying effects on stroke recurrence depending on AF. AF was associated with a fourfold increased risk of stroke recurrence and was an independent predictor of mortality and poor outcomes in patients with ischemic stroke.^36^ Previous studies showed that hypoglycemia was a risk factor for AF^37^ and our subgroup analysis revealed that hypoglycemia might increase recurrence risk in stroke patients with AF due to the close relationship between AF and ischemic stroke. We also found that stress induced hyperglycemia was associated with the stroke recurrence in patients without AF. Previous studies reported a significant association between poor glycemic control and the risk of stroke in patients without AF.^38^ The clinical implication might be that physicians should make individualized glucose control strategies depending on AF. Physicians may pay more attention to intense glucose control in patients without AF, while achieving the target glucose level safely without hypoglycemia and increasing adherence to anticoagulants should be pronounced in patients with AF.

This study had several limitations. First, this was a retrospective study based on a single‐center prospective database, which might generate biases for missing potential confounders and excluding patients with missing baseline HbA1c due to the lower frequency of HbA1c tests conducted in nondiabetic patients. Second, the duration of diagnosis of acute ischemic stroke might affect the interpretation of the results and continuous monitoring of blood glucose might be more informative, despite we tried to avoid bias by limiting the timing of blood sampling to 24 h after admission due to the retrospective nature. Third, the database did not record information on hemoglobin diseases and recent blood transfusions, which might affect the level of HbA1c. Finally, although we provided medications at discharge instead in this study, the dynamic changes and adherence to medical treatments might be more informative. Further prospective cohorts are needed to evaluate the association between the glycemic gap and stroke recurrence in patients with ischemic stroke and verify our findings in different populations.

In conclusion, we found that the glycemic gap was significantly associated with stroke recurrence in patients with ischemic stroke. The glycemic gap was consistently associated with stroke recurrence across subgroups and had varying effects depending on AF.

## FUNDING INFORMATION

The project is supported by Key R&D Program of Jiangsu Province (BE2020700) and National Natural Science Foundation of China (U22A20341 and U20A20357).

## CONFLICT OF INTEREST STATEMENT

The authors have declared no conflicts of interest with respect to the authorship or publication of this article.

## Supporting information


**Data S1.** Supporting Information.
