# Supplementary material for: Association of glycemic gap with stroke recurrence in patients with ischemic stroke
Source: J Diabetes. 2023 Jun 9;15(9):714–23. doi: 10.1111/1753-0407.13432 (PMC10509515; doi:10.1111/1753-0407.13432)
Supplement: Supplementary file 1 — Data S1. Supporting Information. [file JDB-15-714-s001.pdf]

Supplementary Materials

Supplementary Methods

Fractional polynomial model

To account for the possibility of a non-linear relationship, we applied the fractional polynomial[1] terms of the glycemic gap into model 1. The best fitting fractional polynomial model was identified by the multivariable fractional polynomial method. The optimal glycemic gap level was when the first derive of the model was equal to 0. We used the delta method[2] based on standard errors to calculate the confidence intervals for the optimal glycemic gap level.

Bayesian hierarchical logistic regression model

According to the results of subgroup analyses, we hypothesized that the effects of glycemic gaps on stroke recurrence might differ in patients with or without atrial fibrillation. Initially we performed analyses in all patients, then we stratified the cohort by diabetes status to account for the effect of diabetes mellitus on stroke recurrence. To optimize the robustness and eliminate the influence of interaction, we selected the Bayesian hierarchical logistic regression model to explain the likelihood of stroke recurrence at the median follow-up time of 3 years separately in patients with or without atrial fibrillation. Analyses were implemented in Python (version, 3.7.10, "pymc3" package[3]).

Since the coefficients of glycemic gap were estimated in the same model, we could directly quantify the difference of coefficients in patients with or without atrial fibrillation. In line with previous studies[4], we employed the No U-Turn Sampler (NUTS) method, a type of Monte Carlo Markov Chain algorithm (setting: draws = 2000), to draw samples from the posterior predictive distributions and 90% highest probability density interval (HPDI). The differences could be substantial when the reference level of 0 were not included in the range of 90% HPDI. Model specification was presented as follows:

Hyperpriors

$$\sigma\_\beta \sim Halfcauchy(1)$$

$$mu\_\beta \sim Normal(\mu = 0, \sigma = 10)$$

Priors

$$\alpha \sim Normal(\mu = 0, \sigma = 20)$$

$$\beta_{AF,\ Non\text{-}AF} \sim Normal(\mu = mu\_\beta, \sigma = \sigma\_\beta)_{AF,\ non\text{-}AF}$$

Likelihood

$$likelihood = invlogit(\alpha + \beta\ [AF/Non\text{-}AF] * Glycemic\ gap)$$

$$Recurrence\_prob \sim Bernoulli(p = likelihood, observed = Recurrence)$$

Supplementary Table 1. Proportions of characteristics with missing values.

Characteristics	No of patients (%)
BMI	125 (4.6)
NIHSS	6 (0.2)
Smoking status	34 (1.2)
Education	93 (3.4)
WBC	31 (1.1)
Hemoglobin	28 (1.0)
TC	42 (1.5)
TG	41 (1.5)
HDL	100 (3.6)
LDL	94 (3.4)
Creatinine	32 (1.2)
ALT	35 (1.3)

Abbreviations: ALT, alanine aminotransferase; BMI, body mass index; HDL, high density lipoprotein; LDL, low density lipoprotein; NIHSS, National Institute of Health Stroke Scale; TC, total cholesterol; TG, triglyceride; WBC, white blood count.

Supplementary Table 2. Baseline characteristics of patients included and excluded due to missing HbA1c levels.

Characteristics	Excluded<br>N = 1092	Included<br>N = 2734	***P*** **value**
Age, years	58.2 (13.1)	61.7 (11.1)	<0.001
Male, n (%)	792 (72.5)	1924 (70.4)	0.198
BMI, kg/m^2	24.6 (3.5)	24.7 (3.3)	0.671
Baseline NIHSS, score	4.0 [2.0, 9.0]	4.0 [2.0, 9.0]	0.07
Hypertension, n (%)	786 (72.0)	2007 (73.4)	0.39
Diabetes mellitus, n (%)	85 (7.8)	1062 (38.8)	<0.001
Dyslipidemia, n (%)	327 (29.9)	862 (31.5)	0.359
Atrial fibrillation, n (%)	132 (12.1)	296 (10.8)	0.284
Coronary heart disease, n (%)	90 (8.2)	261 (9.5)	0.233
Smoking status, n (%)			0.327
Never	485 (45.2)	1288 (47.1)	
Former	92 (8.6)	254 (9.3)	
Current	496 (46.2)	1192 (43.6)	
Alcohol consumption, n (%)	228 (20.9)	585 (21.4)	
Education, years			0.136
0-6	303 (28.6)	816 (29.8)	
6-9	543 (51.2)	1304 (47.7)	
9-12	101 (9.5)	319 (11.7)	
>12	113 (10.7)	295 (10.8)	
Stroke subtypes, n (%)			0.099
LAA	376 (34.5)	991 (36.2)	
CE	163 (14.9)	354 (12.9)	
SAA	189 (17.3)	530 (19.4)	
SOE	142 (13.0)	298 (10.9)	
SUE	221 (20.3)	561 (20.5)	
Laboratory data			
White blood count, 10^9/L	7.4 [6.1, 9.2]	7.0 [5.8, 8.6]	<0.001
Hemoglobin, g/L	140 [128, 151]	140 [129, 151]	0.765
TC, mmol/L	4.2 [3.6, 5.0]	4.2 [3.6, 5.0]	0.972
TG, mmol/L	1.3 [1.0, 1.9]	1.4 [1.0, 1.9]	0.684
HDL, mmol/L	1.1 (0.3)	1.1 (0.4)	0.861
LDL, mmol/L	3.0 (11.0)	2.7 (1.0)	0.071
Creatine, μmmol/L	65.0 [55.0, 78.0]	64.0 [54.0, 77.0]	0.122
ALT, U/L	18.0 [13.0, 28.0]	17.0 [13.0, 26.0]	0.066

Abbreviations: ALT, alanine aminotransferase; BMI, body mass index; CE, cardio-embolism; CI, confidence interval; HDL, high density lipoprotein; LAA, large-artery atherosclerosis; LDL, low density lipoprotein; NIHSS, National Institute of Health Stroke Scale; OHA, oral hypoglycemic agents; SAA, small-vessel occlusion; SOE, stroke of other determined etiology; SUE, stroke of undetermined etiology; TC, total cholesterol; TG, triglyceride; WBC, white blood count.

Supplementary Table 3. Univariable analysis for stroke recurrence.

Variables	Hazard ratio (95% CI)	*P* value
Age	1.014 (1.005-1.024)	0.004
Male	1.060 (0.849-1.324)	0.605
BMI	0.997 (0.967-1.028)	0.843
Baseline NIHSS score	1.011 (0.996-1.026)	0.152
Hypertension	1.351 (1.058-1.725)	0.016
Diabetes mellitus	1.225 (1.001-1.501)	0.049
Dyslipidemia	1.007 (0.813-1.247)	0.950
Atrial fibrillation	1.509 (1.124-2.027)	0.006
Coronary heart disease	1.162 (0.834-1.618)	0.376
Smoking status		
Never	Reference	
Former	1.106 (0.767-1.594)	0.588
Current	1.239 (1.003-1.531)	0.047
Alcohol consumption	0.626 (0.453-0.865)	0.004
Education		
0-6	Reference	
6-9	0.916 (0.726-1.155)	0.457
9-12	0.882 (0.630-1.237)	0.468
>12	0.797 (0.555-1.144)	0.219
Stroke subtype		
LAA	Reference	
CE	1.174 (0.855-1.611)	0.322
SAA	0.635 (0.464-0.867)	0.004
SOE	1.057 (0.764-1.461)	0.739
SUE	0.937 (0.714-1.230)	0.641
Laboratory data		
WBC	1.023 (0.985-1.063)	0.235
Hemoglobin	0.994 (0.988-1.001)	0.051
TC	0.975 (0.894-1.065)	0.578
TG	0.976 (0.887-1.074)	0.621
HDL	1.026 (0.803-1.311)	0.837
LDL	0.994 (0.898-1.100)	0.903
Glucose	1.037 (0.999-1.077)	0.059
Creatinine	1.002 (0.999-1.004)	0.150
HbA1c	1.044 (0.984-1.107)	0.154
ALT	0.997 (0.992-1.003)	0.345
Prior antidiabetic agents		
None	Reference	
OHA	1.099 (0.741-1.630)	0.637
Insulin	1.161 (0.766-1.759)	0.482
Both	0.912 (0.623-1.335)	0.636
Medications at discharge		

Antiplatelet drugs	0.876 (0.603-1.273)	0.489
Anticoagulants	1.064 (0.704-1.609)	0.769
Statins	0.845 (0.486-1.470)	0.551
Antihypertensive drugs	1.056 (0.863-1.292)	0.597
Hypoglycemic agents	1.249 (1.014-1.538)	0.036

Abbreviations: ALT, alanine aminotransferase; BMI, body mass index; CE, cardio-embolism; CI, confidence interval; HDL, high density lipoprotein; LAA, large-artery atherosclerosis; LDL, low density lipoprotein; NIHSS, National Institute of Health Stroke Scale; OHA, oral hypoglycemic agents; SAA, small-vessel occlusion; SOE, stroke of other determined etiology; SUE, stroke of undetermined etiology; TC, total cholesterol; TG, triglyceride; WBC, white blood count.

Supplementary Table 4. Hazard ratios for all-cause mortality according to glycemic gaps.

		Model 1		Model 2		Model 3	
**Variables**	**No. of events (%)**	**HR (95% CI)**	***P* value**	**HR (95% CI)**	***P* value**	**HR (95% CI)**	***P* value**
Tertiles 1	83/914 (9.08)	1.095 (0.799-1.502)	0.573	0.987 (0.711-1.372)	0.939	0.963 (0.690-1.346)	0.827
Tertiles 2	72/910 (7.91)	Reference		Reference		Reference	
Tertiles 3	150/910 (16.48)	2.304 (1.739-3.052)	<0.001	1.936 (1.454-2.578)	<0.001	1.327 (0.976-1.806)	0.071

Abbreviations: CI, confidence interval; HR, hazard ratio; NIHSS, National Institute of Health Stroke Scale.

Model 1: unadjusted model.

Model 2: adjusted by age, sex, hypertension, diabetes mellitus, atrial fibrillation, dyslipidemia, coronary heart disease, smoking status, alcohol consumption, stroke etiology, education years.

Model 3: adjusted for covariates in model 2 and body mass index, NIHSS, hemoglobin, total cholesterol, triglyceride, high density lipoprotein, low density lipoprotein, prior antidiabetic agents, and the usage of antiplatelet drugs, anticoagulants, antihypertensive drugs and hypoglycemic agents at discharge.

Supplementary Table 5. Odds ratios for favorable outcome according to glycemic gaps.

		Model 1		Model 2		Model 3	
**Variables**	**No. of events (%)**	**OR (95% CI)**	***P* value**	**OR (95% CI)**	***P* value**	**OR (95% CI)**	***P* value**
Tertiles 1	794/914 (86.87)	1.123 (0.860-1.466)	0.395	1.195 (0.895-1.599)	0.228	1.113 (0.804-1.541)	0.520
Tertiles 2	778/910 (85.49)	Reference		Reference		Reference	
Tertiles 3	659/910 (72.42)	0.445 (0.351-0.562)	<0.001	0.491 (0.383-0.628)	<0.001	0.881 (0.659-1.177)	0.391

Abbreviations: CI, confidence interval; OR, odds ratio; NIHSS, National Institute of Health Stroke Scale.

Model 1: unadjusted model.

Model 2: adjusted by age, sex, hypertension, diabetes mellitus, atrial fibrillation, dyslipidemia, coronary heart disease, smoking status, alcohol consumption, stroke etiology, education years.

Model 3: adjusted for covariates in model 2 and body mass index, NIHSS, hemoglobin, total cholesterol, triglyceride, high density lipoprotein, low density lipoprotein, prior antidiabetic agents, and the usage of antiplatelet drugs, anticoagulants, antihypertensive drugs and hypoglycemic agents at discharge.

Supplementary Table 6. Competing risk analysis of glycemic gaps for predicting stroke recurrence.

	Model 1		Model 2		Model 3	
**Variables**	**HR (95% CI)**	***P* value**	**HR (95% CI)**	***P* value**	**HR (95% CI)**	***P* value**
Tertiles 1	1.333 (1.034-1.719)	0.027	1.247 (0.949-1.639)	0.110	1.204 (0.912-1.589)	0.190
Tertiles 2	Reference		Reference		Reference	
Tertiles 3	1.438 (1.116-1.853)	0.005	1.417 (1.094-1.836)	0.008	1.471 (1.128-1.919)	0.004

Abbreviations: CI, confidence interval; HR, hazard ratio; NIHSS, National Institute of Health Stroke Scale.

Model 1: unadjusted model.

Model 2: adjusted by age, sex, hypertension, diabetes mellitus, atrial fibrillation, dyslipidemia, coronary heart disease, smoking status, alcohol consumption, stroke etiology, education years.

Model 3: adjusted for covariates in model 2 and body mass index, NIHSS, hemoglobin, total cholesterol, triglyceride, high density lipoprotein, low density lipoprotein, prior antidiabetic agents, and the usage of antiplatelet drugs, anticoagulants, antihypertensive drugs and hypoglycemic agents at discharge.

Supplementary Table 7. Hazard ratios for stroke recurrence according to DM and AF.

Subgroups	Variables	Model 1 HR (95% CI)	*P* value	Model 2 HR (95% CI)	*P* value	Model 3 HR (95% CI)	*P* value
DM (-) AF (-)	Tertile 1	1.080 (0.733-1.592)	0.697	1.056 (0.714-1.561)	0.786	1.057 (0.704-1.587)	0.788
	Tertile 2	Reference		Reference		Reference	
	Tertile 3	1.461 (1.056-2.022)	0.022	1.458 (1.052-2.019)	0.024	1.422 (1.015-1.992)	0.041
DM (+) AF (-)	Tertile 1	1.066 (0.662-1.715)	0.794	1.137 (0.702-1.839)	0.602	1.077 (0.661-1.757)	0.765
	Tertile 2	Reference		Reference		Reference	
	Tertile 3	1.448 (0.874-2.399)	0.150	1.464 (0.880-2.435)	0.142	1.467 (0.871-2.470)	0.150
DM (-) AF (+)	Tertile 1	3.746 (1.317-10.653)	0.013	3.441 (1.129-10.49)	0.030	3.583 (1.112-11.444)	0.031
	Tertile 2	Reference		Reference		Reference	
	Tertile 3	2.036 (0.733-5.658)	0.173	1.903 (0.676-5.359)	0.223	1.681 (0.550-5.153)	0.361
DM (+) AF (+)	Tertile 1	3.119 (0.703-13.84)	0.135	2.56 (0.546-12.005)	0.233	36.779 (14.250-94.927)	<0.001
	Tertile 2	Reference		Reference		Reference	
	Tertile 3	1.439 (0.279-7.427)	0.664	1.259 (0.235-6.738)	0.788	0.464 (0.165-1.305)	0.146

Abbreviations: AF, atrial fibrillation; CI, confidence interval; DM, diabetes mellitus; HR, hazard ratio; NIHSS, National Institute of Health Stroke Scale.

Model 1: unadjusted model.

Model 2: adjusted by age, sex, hypertension, dyslipidemia, coronary heart disease, smoking status, alcohol consumption, stroke etiology, education years.

Model 3: adjusted for covariates in model 2 and body mass index, NIHSS, hemoglobin, total cholesterol, triglyceride, high density lipoprotein, low density lipoprotein, prior antidiabetic agents, and the usage of antiplatelet drugs, anticoagulants, antihypertensive drugs and hypoglycemic agents at discharge.

Supplementary Figure 1. Study flowchart.

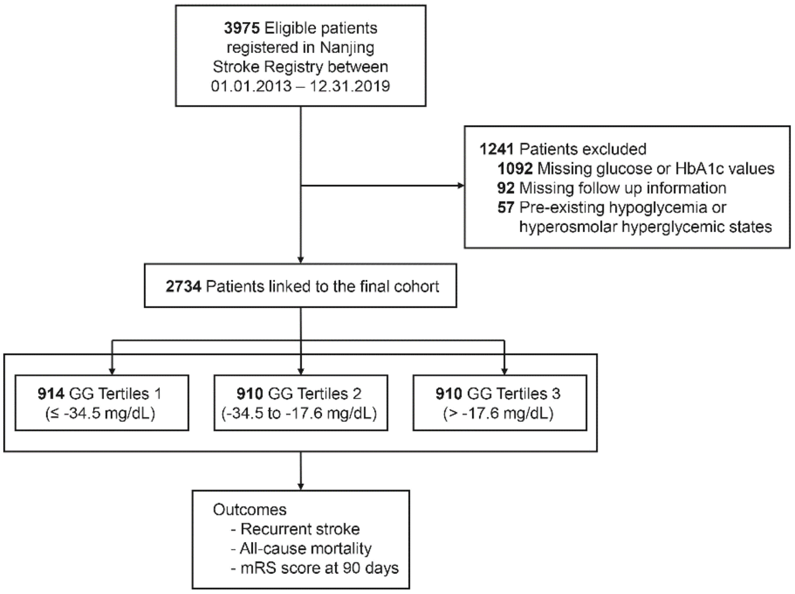

Abbreviations: GG, glycemic gap; mRS, modified Rankin Scale.

Supplementary Figure 2. Distribution of modified Rankin Scale at 90 days according to glycemic gap tertiles.

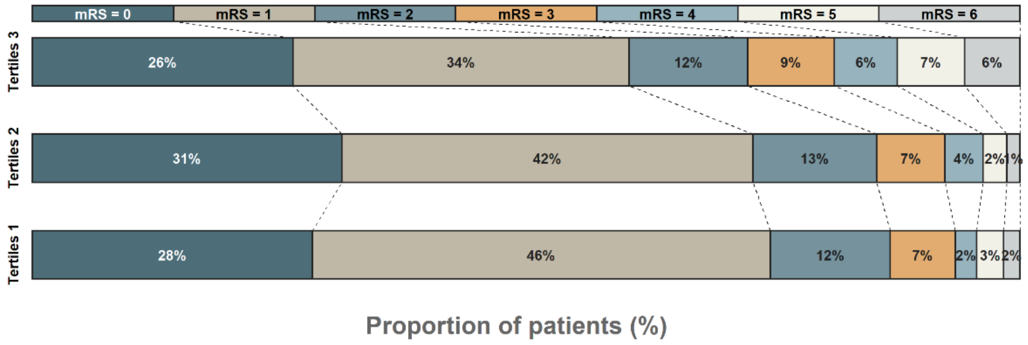

Abbreviations: mRS, modified Rankin Scale.

Supplementary Figure 3. Association of glycemic gap and glucose with favorable outcomes in patients with ischemic stroke.

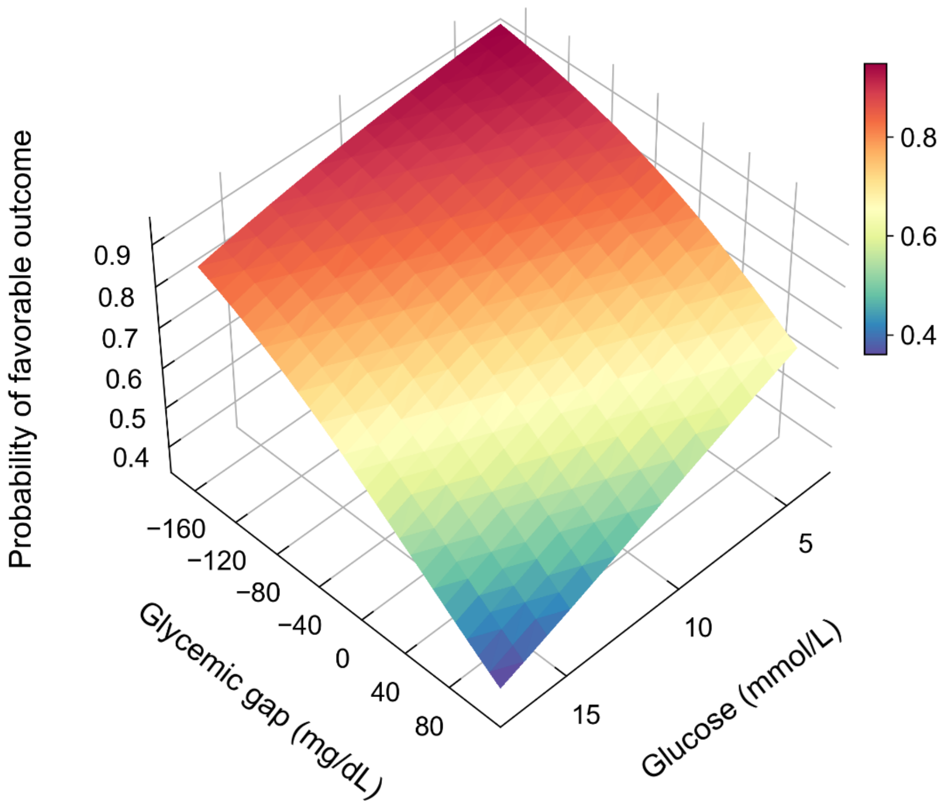

Supplementary Figure 4. Subgroup analysis to investigate the association between glycemic gap and stroke recurrence in patients with ischemic stroke according to diabetes mellitus and atrial fibrillation statuses.

Models	Levels	HR (95% CI)	*P* value
**Model 1**			
Non-DM	Tertiles 1	1.298 (0.912-1.846)	0.147
	Tertiles 3	1.503 (1.107-2.041)	0.009
DM	Tertiles 1	1.175 (0.748-1.845)	0.485
	Tertiles 3	1.446 (0.892-2.342)	0.134
AF	Tertiles 1	3.706 (1.599-8.586)	0.002
	Tertiles 3	1.842 (0.774-4.384)	0.167
Non-AF	Tertiles 1	1.162 (0.886-1.524)	0.279
	Tertiles 3	1.501 (1.147-1.963)	0.003
**Model 2**			
Non-DM	Tertiles 1	1.262 (0.885-1.799)	0.199
	Tertiles 3	1.510 (1.108-2.058)	0.009
DM	Tertiles 1	1.272 (0.804-2.014)	0.304
	Tertiles 3	1.461 (0.896-2.381)	0.129
AF	Tertiles 1	4.103 (1.692-9.947)	0.002
	Tertiles 3	1.767 (0.729-4.284)	0.207
Non-AF	Tertiles 1	1.150 (0.875-1.512)	0.315
	Tertiles 3	1.535 (1.171-2.012)	0.002
**Model 3**			
Non-DM	Tertiles 1	1.260 (0.873-1.819)	0.217
	Tertiles 3	1.490 (1.080-2.056)	0.015
DM	Tertiles 1	1.267 (0.794-2.021)	0.321
	Tertiles 3	1.493 (0.904-2.465)	0.117
AF	Tertiles 1	4.403 (1.742-11.127)	0.002
	Tertiles 3	2.220 (0.851-5.789)	0.103
Non-AF	Tertiles 1	1.060 (0.792-1.418)	0.697
	Tertiles 3	1.512 (1.143-2.001)	0.004

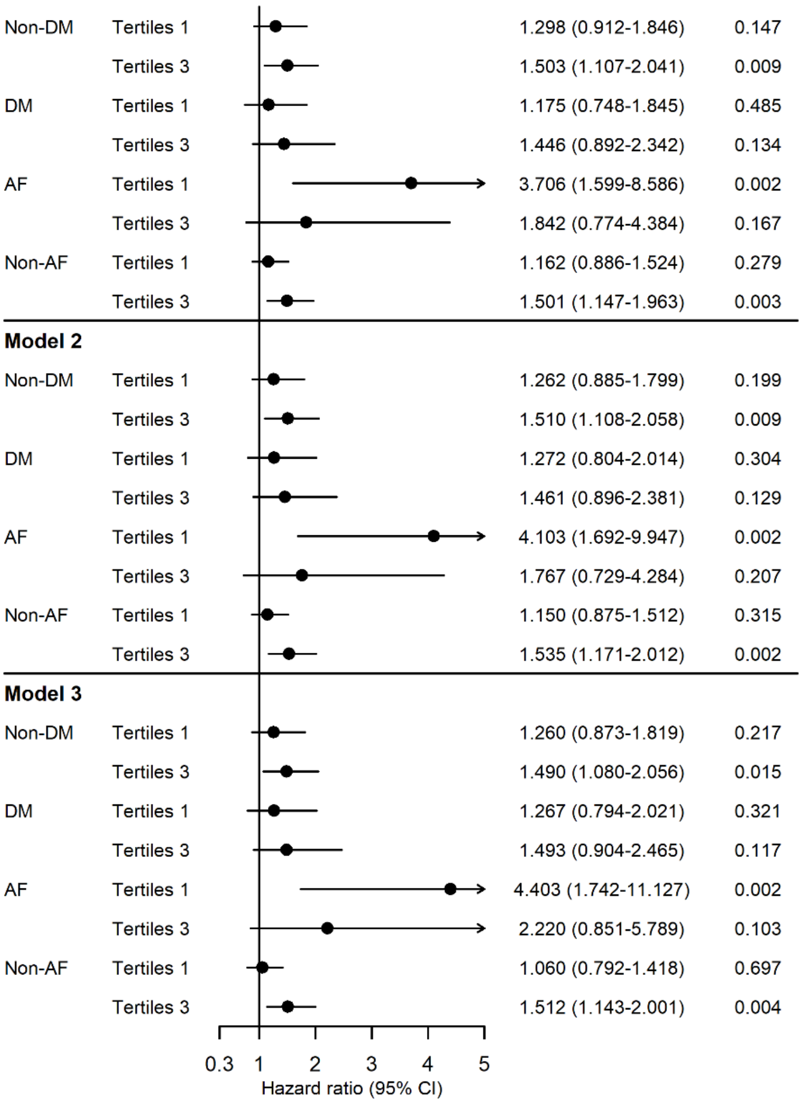

Abbreviations: AF, atrial fibrillation; CI, confidence interval; DM, diabetes mellitus; HR, hazard ratio; NIHSS, National Institute of Health Stroke Scale.

Model 1: unadjusted model.

Model 2: adjusted by age, sex, hypertension, dyslipidemia, coronary heart disease, smoking status, alcohol consumption, stroke etiology, education years.

Model 3: adjusted for covariates in model 2 and body mass index, NIHSS, hemoglobin, total cholesterol, triglyceride, high density lipoprotein, low density lipoprotein, prior antidiabetic agents, and the usage of antiplatelet drugs, anticoagulants, antihypertensive drugs and hypoglycemic agents at discharge.

References

1. Royston P, Sauerbrei W. A new approach to modelling interactions between treatment and continuous covariates in clinical trials by using fractional polynomials. Stat Med. 2004;23:2509–2525.
2. Lye J, Hirschberg J. Confidence Intervals for Ratios: Econometric Examples in Stata and R. :83.
3. Salvatier J, Wiecki TV, Fonnesbeck C. Probabilistic programming in Python using PyMC3. PeerJ Comput. Sci. 2016;2:e55.
4. Bonkhoff AK, Schirmer MD, Bretzner M, Hong S, Regenhardt RW, Brudfors M, Donahue KL, Nardin MJ, Dalca AV, Giese A-K, et al. Outcome after acute ischemic stroke is linked to sex-specific lesion patterns. Nat Commun. 2021;12:3289.
